# The Inversion In(2L)t Impacts Complex, Temperature‐Sensitive Behaviors in *Drosophila melanogaster*

**DOI:** 10.1002/ece3.74029

**Published:** 2026-08-17

**Authors:** Benedict Adam Lenhart, Alan Olav Bergland

**Affiliations:** ^1^ Department of Genome Science University of Virginia Charlottesville Virginia USA; ^2^ Department of Biology University of Virginia Charlottesville Virginia USA

**Keywords:** activity, behavior, *Drosophila melanogaster*, inversion

## Abstract

Genetic variation in behavioral traits allows organisms to respond and adapt to environmental challenges. Genetic variation in behavior is often affected by many genes and thus has a complex genetic basis. Inversions, the reorientation of genes along the chromosome, tightly link genetic variants together because they suppress recombination. Therefore, inversions are believed to have a major impact on phenotypic variation because they combine the effects of multiple genes, which can pleiotropically alter multiple aspects of behavior. This study investigates how the inversion In(2L)t, found in *Drosophila melanogaster* populations around the world, impacts different aspects of behavior in an environment‐sensitive manner. We test the activity, locomotion, and startle‐induced behavior of flies with different In(2L)t genotypes across sex and temperatures. We observe that *Drosophila* homozygous for In(2L)t spend more time active, spend more time away from a source of food, and have a smaller intensity of startle response than flies heterozygous for the inversion or homozygous for the standard arrangement. Additionally, the impacts of In(2L)t on aspects of behavior can be sex‐specific and are largely consistent across temperatures. Taken together, our research demonstrates that inversions can regulate aspects of behavior, and suggests hypotheses explaining the distribution of In(2L)t across space and time.

## Graphical abstract

The genomic inversion In(2L)t has been shown to alter many phenotypes in *Drosophila melangaster* in a manner thought to mediates melanogaster's seasonal adaptation. We use modern high‐throughput tools to demonstrate that In(2L)t genotype has pronounced impact of areas of melanogaster behavior including activity, startle response, and exploration around a food source. This finding further validates the study of In(2L)t as a genomic feature involved in seasonal evolution, and implicates genes within the inversion for targets of future study.

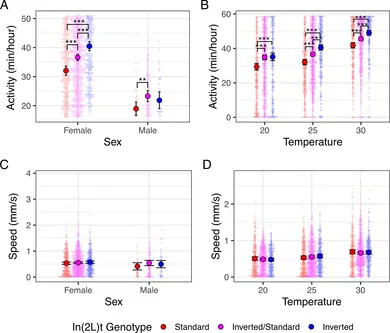

## 1 Introduction

Animals adjust their behavior in response to environmental change that occurs on multiple timescales. Long‐term environmental shifts, such as climate change, influence habitat use and the timing of reproduction (Beever et al. 2017; Miller‐Rushing et al. 2008), while short‐term environmental changes, such as the day/night cycle, affect foraging and activity levels (Ashby 1972; Beauchamp 2007). Some behavioral changes are cyclical, like the seasonal changes in migratory behavior (Chapman et al. 2015) or mating behavior (Riters and Stevenson 2022). In general, behavioral variation is considered plastic in that a single individual modifies its behavior in response to environmental change (Snell‐Rood 2013). Natural populations exhibit genetic variation in this behavioral plasticity, which in turn influences both baseline behaviors (Fleury et al. 1995; Wong et al. 2019) and how behavior responds to environmental shifts (Flint 2003; Niepoth and Bendesky 2020).

Advancements in technology have improved our ability to quantify genetic and environmental effects on behavior (Pereira et al. 2020), and to do so at large scale (Schaefer and Claridge‐Chang 2012). Early methods to track *Drosophila* behavior required manual observation of activity, either visually (Kernan et al. 1994; Stacey et al. 2010), or from recordings (Gargano et al. 2005). Measurement of larger populations became possible with beam‐break systems that inferred circadian activity and sleep (Chiu et al. 2010; Cichewicz and Hirsh 2018; Pfeiffenberger et al. 2010). Later, video tracking software allowed quantification of speed (Faville et al. 2015), spatial positioning (Donelson et al. 2012), and responses to sensory stimuli (Werkhoven et al. 2019) often at scale (Elya et al. 2023; Okuyama et al. 2025; Sato and Takahashi 2025; Werkhoven et al. 2019, 2021).

The ubiquity of phenotypic and genotypic variation (David and Capy 1988; Mackay and Huang 2018), coupled with the ease of genetics and experimental study makes *D. melanogaster* a tractable model for studies into the natural genetic variation in behavior. One particularly interesting genetic variant is the large (10 Mb) structural inversion, In(2L)t. Structural inversions such as In(2L)t suppress recombination for genes in and near the inverted region (Charlesworth and Charlesworth 1973; Dobzhansky 1937), and can thus maintain adaptive alleles that impact multiple traits (Kirkpatrick and Barton 2006). The In(2L)t inversion is linked to elevated startle response duration (Mackay et al. 2012; Nunez et al. 2024), foraging behavior (Lee et al. 2017), and many other traits (Lenhart and Bergland 2026). In(2L)t is found at intermediate frequencies in populations worldwide (Nunez et al. 2025; Stalker 1980; van Delden and Kamping 1989) and appears to mediate seasonal adaption in *D. melanogaster* as its frequency shifts seasonally (Nunez et al. 2024). Previous studies that report the behavioral impact of In(2L)t used the beam‐break model or other methods with large‐grain temporal sampling (Lee et al. 2017; Mackay et al. 2012; Nunez et al. 2024). As previous studies only examine one environment, there is opportunity for better characterization of In(2L)t's impact on fine‐scale behavioral plasticity across a range of environments.

To explore how In(2L)t alters fine‐scale behavior and stimulus response in an environment‐specific manner, we employed automated behavioral tracking. We generated F1 offspring with different In(2L)t genotypes through controlled crosses and measured aspects of behavior of flies experiencing different temperatures. We found that In(2L)t is associated with increases in the duration of startle response, the time spent active, and proximity to food. Furthermore, these inversion‐driven differences often vary in intensity across sex while being largely consistent across temperatures.

## 2 Methods

### 2.1 Fly Stocks and Husbandry

We measured the behavior of F1s, generated through controlled crosses between inbred strains of the *Drosophila* Genetic Reference Panel (DGRP) (Mackay et al. 2012). By crossing five lines homozygous for In(2L)t (DGRP: 32,161, 348, 386, 837) and four lines homozygous for the “standard” gene arrangement (DGRP: 57, 189, 634, 853), we generated 8 unique F1 crosses homozygous for In(2L)t, 16 crosses heterozygous for In(2L)t, and 6 crosses homozygous for the standard genotype (Table S1). Note that only a subset of possible crosses were used in this study, and a portion of these crosses possess other cosmopolitan inversions such as In(3R)Mo. Flies were kept on standard media from Archon Scientific (Durham, NC; food is composed of 86% water, 0.574% agar, 6.30% cornmeal, 1.52% yeast, 4.65% molasses, 0.39% propionic acid, 0.15% methylparaben, and 0.52% ethanol). Flies were reared from egg to adult in a 25°C incubator set to a 12H:12H light/dark schedule and 50% relative humidity. For each behavioral measurement, we used 2–5 day old non‐virgin flies.

### 2.2 Behavioral Assays

We recorded the behavior of the F1 offspring using the *Drosophila* Arousal Threshold (DART) device (BFK Labs, Hertford, UK; Faville et al. 2015) set on top of an antivibration marble table. Flies were placed within plastic tubes with a 1.5% agar, 5% sucrose solution to prevent desiccation and starvation and a cotton plug to prevent asphyxiation. Before assays, we acclimated the flies in the DART overnight. The flies were kept in constant darkness, 50% relative humidity, and at three temperatures (20°C, 25°C, and 30°C). We recorded the basal activity and induced activity using mechanical stimulus with a built‐in motor. The DART's motor was set to default (~200 hz, five 200 ms pulses, separated by 800 ms). Fly behavior was observed for at least 1 h prior and post each stimulus, with at least 2–3 startle events performed per individual. We performed this experiment in three experimental blocks, each time performing new DGRP crosses (Table S1) and using the same behavioral quantification methods. The first experiment block (414 F1 individuals) was intended to test if the inversion impacted behavior. After identifying a much stronger effect of inversion in females, the second (693 individuals) and third experiment block (709 individuals) surveyed female flies across a broader spectrum of natural genetic variation. Within each experiment block, data collection occurred over multiple days with different batches of flies to accommodate the DART's 108 fly maximum. We measured male and female fly behavior at 25°C and then compared the behavior of female flies at 20°C, 25°C and 30°C.

### 2.3 Behavioral Quantification

We used the DART MATLAB software (version 811f772; Faville et al. 2015) to track the motion of individual flies and quantify their behavior. We focused on two aspects of baseline behavior: duration of time spent active (minutes per hour, m/h) and average speed (millimeters per second, mm/s). We assessed the activity phenotype by first identifying bouts of inactivity defined as at least 5 min spent stationary and then calculating the average time in minutes per hour the fly remained active. We assessed the baseline speed of the fly as the average millimeters moved per second in the hour prior to first stimulus.

We characterized fly startle response by measuring the duration of time with increased speed post‐stimulus and the magnitude of increased speed post‐stimulus. We first established individual fly baseline speed as the average speed in millimeters/s in the hour before the first stimulus, then estimated the duration of startle induced speed change as the time from the stimulus until the fly's speed returned to its baseline speed. To estimate the magnitude of speed increase, we calculated the difference in speed in the minute after the stimulus from the baseline speed, including reductions in speed relative to baseline. Startle response metrics were calculated regardless of whether the fly was inactive prior to stimulus.

To assess the fly's locomotion in relation to their food source, we divided each tube into eight equal‐length regions and calculated the proportion of time spent in the near food‐region and farthest from food region compared to the other seven regions.

### 2.4 Statistical Analysis

We analyzed the impact of sex on behavioral traits using model comparison to identify the impact of inversion genotype, sex, as well as to test for interaction between inversion and sex. This analysis compared the observances of male and female flies exclusively at 25°C. We created the following models:

Null model:Trait~experiment+experiment:day

$$\text{Inversion model}:\text{Trait}\sim \text{In}\left(\text{2L}\right)+\text{experiment}+\text{experiment}:\mathrm{day}$$

$$\mathrm{Sex}\;\text{model}:\text{Trait}\sim \text{In}\left(\text{2L}\right)t+\mathrm{sex}+\text{experiment}+\text{experiment}:\mathrm{day}$$

$$\begin{array}{c} \text{Interaction model}:\text{Trait}\sim \text{In}\left(\text{2L}\right)t+\mathrm{sex}+\text{In}\left(\text{2L}\right)t^{*}\;\mathrm{sex}\\ +\text{experiment}+\text{experiment}:\mathrm{day} \end{array}$$

where In(2L)t is the fixed effect of inversion genotype, *sex* is the fixed effect of sex, *experiment* is a random effect of the experimental block, and *experiment*: day is the random effect of day‐of‐data‐collection nested within the given experiment. Linear mixed‐effects models (LMMs) were fit using maximum‐likelihood implemented in *lme4* (version 1.1‐34, Bates et al. 2015) using a Gaussian error distribution, identity link. We compared the models using the *anova()* function from R version 4.3.1 to estimate the statistical significance of each of the fixed effects using sequential likelihood ratio tests. To analyze the proportion of time flies spent near food, we first transformed proportions with an arcsin‐square root transformation.

Next, we used model comparison to identify the impact of inversion genotype, temperature, as well as to test for gene‐by‐environment interaction between inversion and temperature. This analysis exclusively used data from female flies observed at 20°C, 25°C, and 30°C. We created the following models:

Null model:Trait~experiment+experiment:day

$$\text{Inversion model}:\text{Trait}\sim \text{In}\left(\text{2L}\right)+\text{experiment}+\text{experiment}:\mathrm{day}$$

$$\begin{array}{c} \text{Temperature model}:\text{Trait}\sim \text{In}\left(\text{2L}\right)t+\text{temperature}\\ +\text{experiment}+\text{experiment}:\mathrm{day} \end{array}$$

$$\begin{array}{c} \text{Interaction model}:\text{Trait}\sim \text{In}\left(\text{2L}\right)t+\text{temperature}\\ +\text{In}\left(\text{2L}\right)t^{*}\;\text{temperature}+\text{experiment}+\text{experiment}:\mathrm{day} \end{array}$$

where the *temperature* term is the fixed effect of temperature in Celsius of the environmental chamber, and the other terms are the same as above.

In both sets of models—either examining genotype and sex or genotype and temperature—if a significant effect was found using model comparison for the fixed effects of genotype or an interaction term including genotype, post hoc pairwise Student's *t*‐tests were used to find differences between pairs of genotypes within sex or temperature. Pairwise *t*‐test results were adjusted for multiple testing within phenotype families (every comparison within a trait) using Benjamin‐Hochburg FDR.

## 3 Results

### 3.1 In(2L)t Affects Baseline Levels of Activity

**FIGURE 1:**
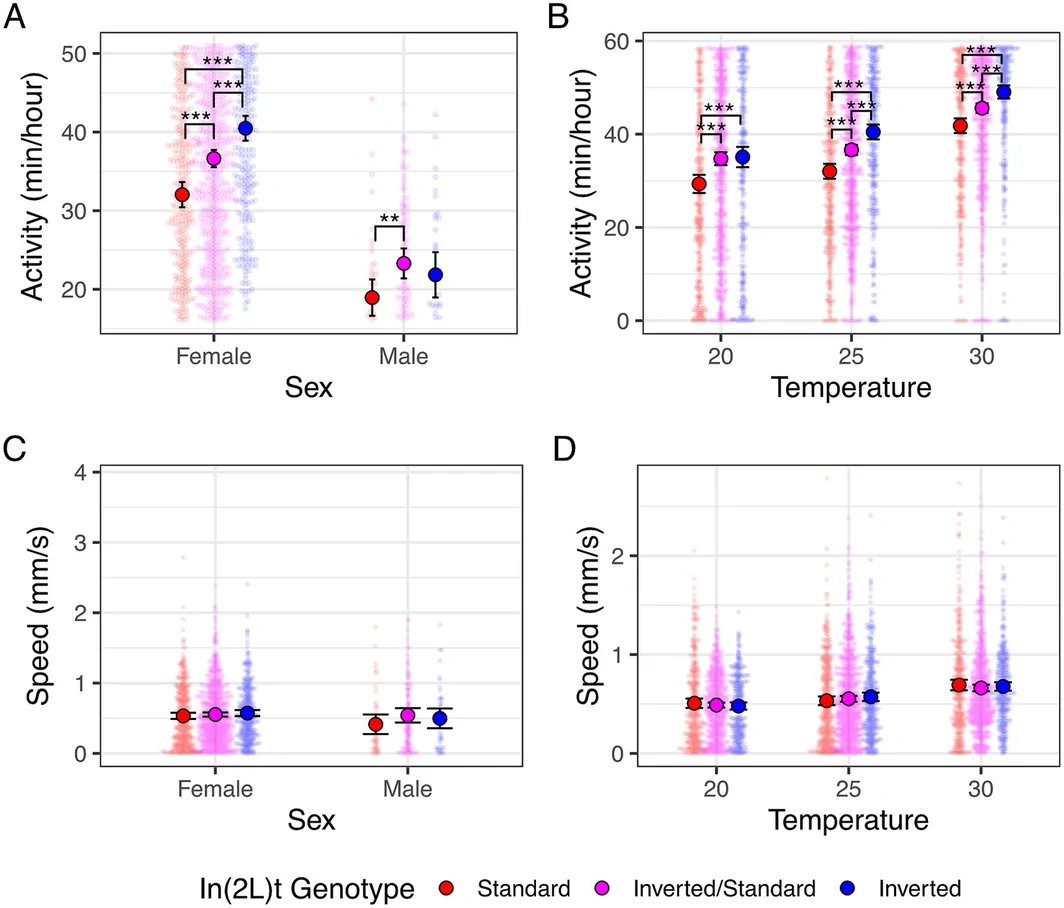
In(2L)t presence is associated with more time active. (A) Measurements of the minutes spent active per hour across sexes at 25°C, colored by the In(2L)t genotype. Large circle and bars show mean and 95% confidence intervals, smaller dots are the individual‐level data. (B) Comparison of time spent active over a range of temperatures for females. (C) Baseline speed at 25°C measured in mm/s across sexes, colored by presence of In(2L)t. (D) Same as in C, but comparing across temperatures. In all plots, the large circles represent the means, error bars represent the 95% confidence intervals (*p < 0.05, **p < 0.01, ***p < 0.001, no *p ≥ 0.05), and small points represent individual level data. Female measurements shown in panels A and C are redrawn in panels B and D, respectively, at 25°C. Individual data points at activity = 60 min/h are condensed for plot clarity.

The amount of time flies spend active is significantly affected by inversion genotype ($\text{Likelihood Ratio Test}:\chi^{2}$ = 68.27, df = 2, *p* = 1.49e‐15; Table 1; Figure 1A) when comparing the sexes at 25°C, with standard genotype flies and males active for less time on average. Female flies homozygous for the inversion are active for longer than heterozygous flies (*t*‐test: *t* = 3.93, df = 743, FDR corrected *p* = 1.7e‐4), and heterozygote females are active for longer than homozygous standard females (*t* = −4.65, df = 811, FDR corrected *p* = 1.53e‐5; Figure 1A). Male flies homozygous for the inversion are also active longer than heterozygotes (*t* = −2.9, df = 106, FDR corrected *p* = 0.006). Sex has a significant effect on time spent active (*χ* ^2^ = 44.89, df = 1, *p* = 2.08e‐11), but there is no interaction between sex and inversion genotype ($\chi^{2}$ = 3.6, df = 2, *p* = 0.17).

**TABLE 1:** Summary statistics from model comparison of flies of both sexes. Comparison of null, inversion, sex, and interaction is given for each of the 6 traits reported on.

Trait	Model	npar	AIC	logLik	Chisq	df	Pr(>Chisq)
Activity	Null model	4	14,747	−7370			
Activity	Inversion model	6	14,683	−7335	68.27	2	1.49E‐15
Activity	Sex model	7	14,640	−7313	44.89	1	2.08E‐11
Activity	Interaction model	9	14,640	−7311	3.60	2	1.66E‐01
Base speed	Null model	4	1727	−860			
Base speed	Inversion model	6	1726	−857	4.92	2	8.52E‐02
Base speed	Sex model	7	1727	−856	1.54	1	2.15E‐01
Base speed	Interaction model	9	1730	−856	1.24	2	5.38E‐01
Startle duration	Null model	4	−2728	1368			
Startle duration	Inversion model	6	−2724	1368	0.35	2	8.41E‐01
Startle duration	Sex model	7	−2724	1369	2.40	1	1.21E‐01
Startle duration	Interaction model	9	−2721	1369	0.51	2	7.75E‐01
Startle magnitude	Null model	4	3513	−1752			
Startle magnitude	Inversion model	6	3513	−1750	3.94	2	1.40E‐01
Startle magnitude	Sex model	7	3509	−1748	5.12	1	2.36E‐02
Startle magnitude	Interaction model	9	3512	−1747	1.06	2	5.89E‐01
%Time near food	Null model	4	−621	314			
%Time near food	Inversion model	6	−622	317	5.71	2	5.76E‐02
%Time near food	Sex model	7	−620	317	0.16	1	6.91E‐01
%Time near food	Interaction model	9	−617	317	0.24	2	8.88E‐01
%Time distant from food	Null model	4	−280	144			
%Time distant from food	Inversion model	6	−313	163	37.80	2	6.18E‐09
%Time distant from food	Sex model	7	−330	172	18.39	1	1.80E‐05
%Time distant from food	Interaction model	9	−326	172	0.28	2	8.71E‐01

Inversion genotype also significantly impacts time spent active when considering females across temperatures ($\chi^{2}$ = 103.05, df = 2, *p* < 2e‐16; Table 2). At 20°C, heterozygous females are as active as inverted homozygous females (*t* = −0.27, df = 606, FDR corrected *p* = 0.78: Figure 1B), suggesting that the inverted allele is dominant, whereas at warmer temperatures (30 degrees) the heterozygotes had an intermediate amount of time active, with inverted homozygous flies active longer than standard flies (*t* = −6.74, df = 709, FDR corrected *p* = 2.00e‐10). Temperature significantly impacts time spent active, with flies active more at higher temperatures ($\chi^{2}$ = 425.74, df = 1, *p* < 2e‐16; Table 1). In general, differences in activity between genotypes were preserved across temperatures (Figure 1B), and we did not observe gene‐by‐environment interaction ($\chi^{2}$ = 5.11, df = 2, *p* = 0.078).

**TABLE 2:** Summary statistics from model comparison of female flies across temperatures. Comparison of null, inversion, sex, and interaction is given for each of the 6 traits reported on.

Trait	Model	npar	AIC	logLik	Chisq	df	Pr(>Chisq)
Activity	Null model	4	37,095	−18,544			
Activity	Inversion model	6	36,996	−18,492	103.05	2	4.19E‐23
Activity	Temp model	7	36,572	−18,279	425.74	1	1.37E‐94
Activity	Interaction model	9	36,571	−18,277	5.11	2	7.78E‐02
Base speed	Null model	4	4077	−2035			
Base speed	Inversion model	6	4080	−2034	1.02	2	6.00E‐01
Base speed	Temp model	7	3952	−1969	130.81	1	2.72E‐30
Base speed	Interaction model	9	3955	−1969	0.44	2	8.03E‐01
Startle duration	Null model	4	−6056	3032			
Startle duration	Inversion model	6	−6052	3032	0.26	2	8.80E‐01
Startle duration	Temp model	7	−6140	3077	89.71	1	2.75E‐21
Startle duration	Interaction model	9	−6137	3078	1.40	2	4.96E‐01
Startle magnitude	Null model	4	8228	−4110			
Startle magnitude	Inversion model	6	8221	−4104	11.09	2	3.90E‐03
Startle magnitude	Temp model	7	8121	−4054	101.80	1	6.14E‐24
Startle magnitude	Interaction model	9	8117	−4049	8.65	2	1.32E‐02
%Time near food	Null model	4	−1275	642			
%Time near food	Inversion model	6	−1294	653	22.90	2	1.07E‐05
%Time near food	Temp model	7	−1479	746	186.82	1	1.57E‐42
%Time near food	Interaction model	9	−1475	747	0.42	2	8.11E‐01
%Time distant from food	Null model	4	−648	328			
%Time distant from food	Inversion model	6	−722	367	78.01	2	1.15E‐17
%Time distant from food	Temp model	7	−722	368	2.07	1	1.50E‐01
%Time distant from food	Interaction model	9	−721	369	2.37	2	3.06E‐01

We did not observe a significant difference in base speed between genotypes ($\chi^{2}$ = 4.92, df = 2, *p* = 0.085; Table 1; Figure 1C), sexes ($\chi^{2}$ = 1.54, df = 1, *p* = 0.21), or any interaction between genotype and sex ($\chi^{2}$ = 1.24, df = 2, *p* = 0.54) at 25°C. The inversion does not affect base speed in females across temperatures ($\chi^{2}$ = 1.02, df = 2, *p* = 0.6). However, there was a significant effect of temperature on speed with flies moving faster in warmer environments ($\chi^{2}$= 130.81, df = 2, *p* = <2e‐16; Table 2; Figure 1D). There is no gene‐by‐environment interaction for speed ($\chi^{2}$= 0.44, df = 2, *p* = 0.80).

### 3.2 Inversion Genotype Affects Startle Response Intensity

**FIGURE 2:**
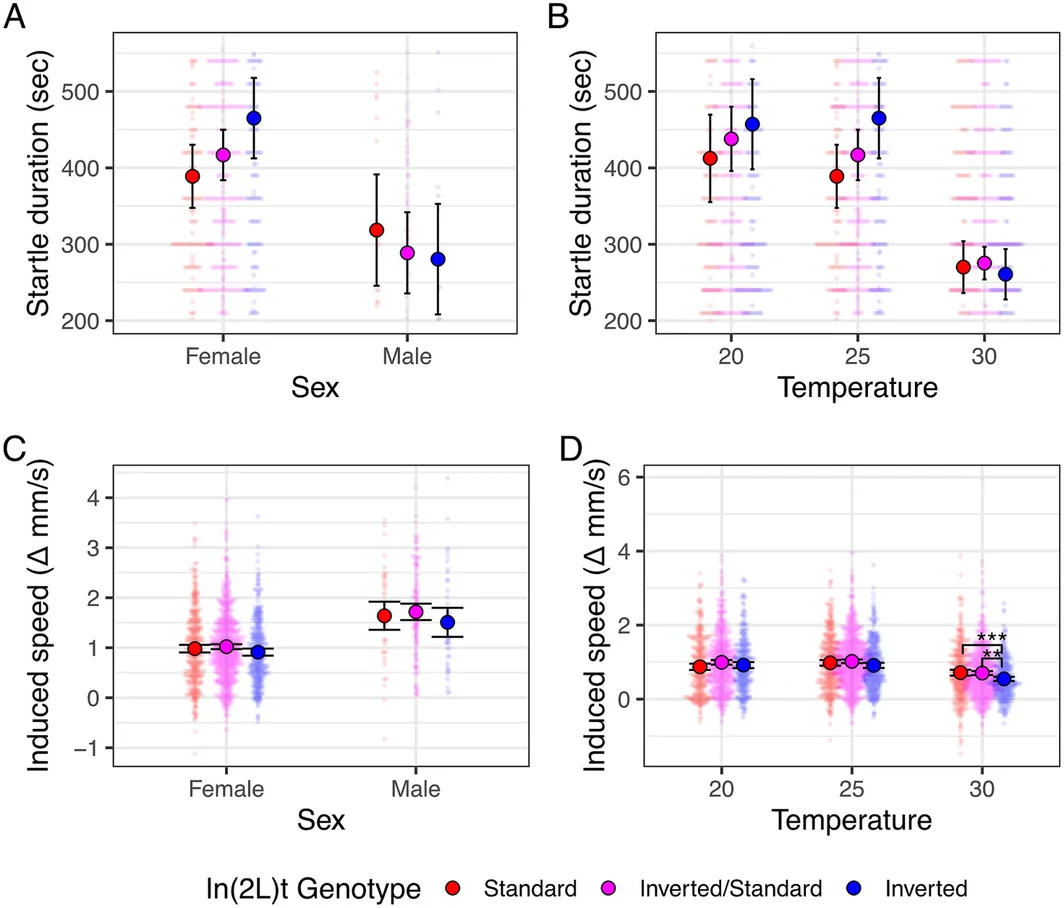
Inverted homozygous flies exhibit differences in startle induced speed. (A) Startle response at 25°C measured as the duration of elevated response relative to baseline, colored by In(2L)t presence. (B) Same as in A, but across temperatures. (C) Induced activity at 25°C, defined as the difference in speed between startled state and baseline, again colored by In(2L)t genotype. (D) Same as in C, but across temperatures. In all plots, the large circles represent the means, error bars represent the 95% confidence intervals (*p < 0.05, **p < 0.01, ***p < 0.001, no *p ≥ 0.05), and small points represent individual level data. Female measurements shown in panels A and C are redrawn in panels B and D, respectively, at 25°C.

There is no effect of inversion genotype (*χ* ^2^ = 0.35, df = 2, *p* = 0.84), sex (*χ* ^2^ = 2.4, df = 1, *p* = 0.12; Table 1, Figure 2A), nor inversion‐sex interaction (*χ* ^2^ = 0.51, df = 2, *p* = 0.78) on startle‐induced changes in activity duration. When considering females across temperatures, there is no effect of genotype on the duration of startle response (*χ* ^2^ = 0.26, df = 2, *p* = 0.88; Table 2; Figure 2B). Temperature does impact the startle response duration (*χ* ^2^ = 88.71, df = 1, *p* < 2e‐16), but there is no observed gene‐by‐environment interaction (*χ* ^2^ = 1.4, df = 2, *p* = 0.50).

Inversion genotype of the flies overall does not impact changes in speed following a startle (*χ* ^2^ = 3.9, df = 2, *p* = 0.14; Table 1; Figure 2C). The sex of the flies significantly impacts startle‐induced speed (*χ* ^2^ = 5.12, df = 1, *p* = 0.024), with male flies showing a larger response, and there is no observed genotype‐sex interaction (*χ* ^2^ = 1.06, df = 2, *p* = 0.59).

However, when considering female flies across temperatures, In(2L)t significantly impacts changes in speed following startle (*χ* ^2^ = 11.09, df = 2, *p* = 0.0039; Table 2; Figure 2D). Interestingly, genotype differences are only observed at warm temperatures, where inverted homozygotes have lower induced speed at 30°C than heterozygotes (*t* = −4.04, df = 790, FDR corrected *p* = 7.1e‐4) or standard flies (*t* = 3.41, df = 612, FDR corrected *p* = 4.2e‐3; Figure 2D). Not only does temperature impact the fly speed following startle (*χ* ^2^ = 101.8, df = 1, *p* < 2e‐16), there is a gene‐by‐environment interaction observed in that the inverted genotype decreases in its response compared to the other genotypes as temperature increases (*χ* ^2^ = 8.65, df = 2, *p* = 0.013).

### 3.3 In(2L)t Is Associated With Location in Relation to Food

**FIGURE 3:**
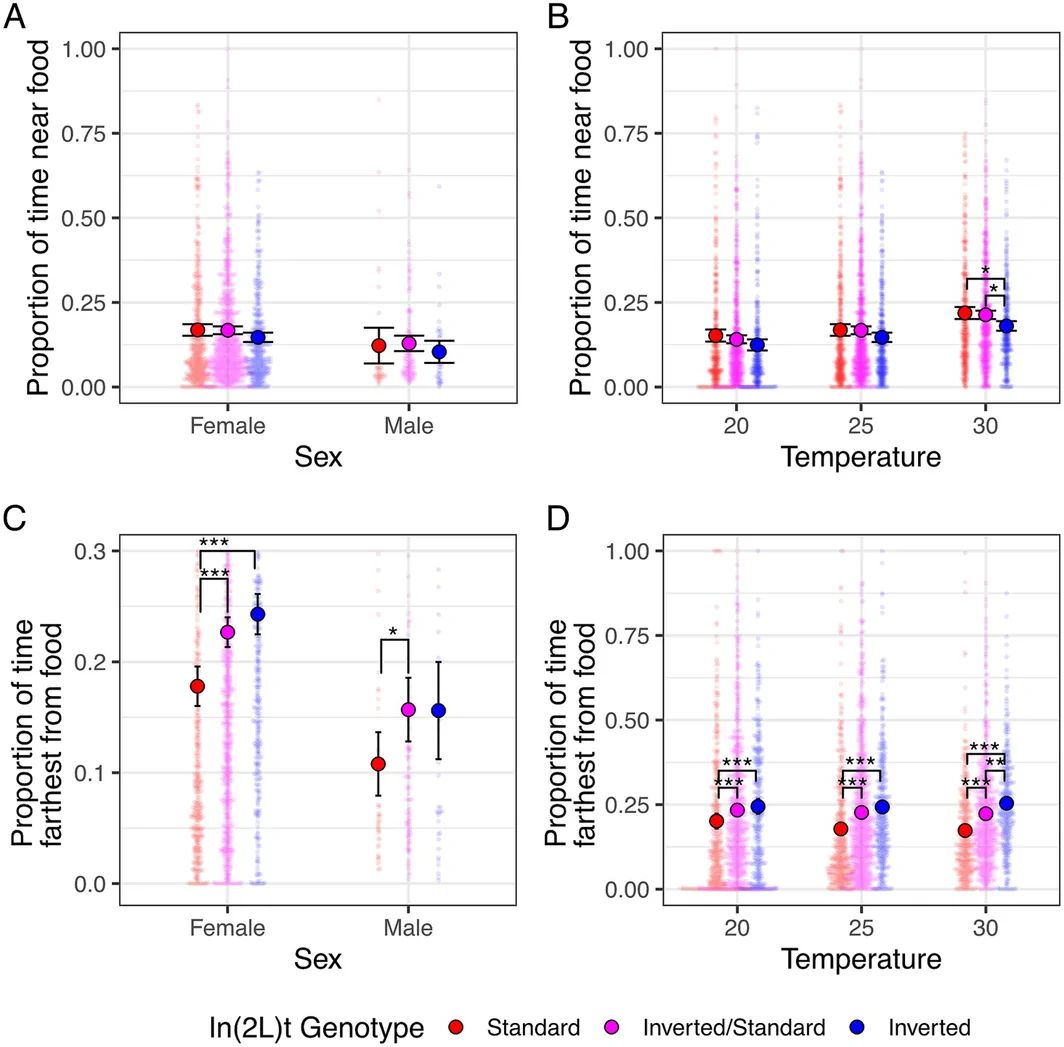
Inverted homozygous flies spend more time away from food source. (A) Proportion of time spent in the region nearest food at 25°C within the DART experiment, colored by presence of In(2L)t. (B) Same as in A, but across temperature. (C) Proportion of time spent in the region farthest from food at 25°C within the DART experiment, colored by presence of In(2L)t. (D) Same as in C, but across temperature. In all plots, the large circles represent the means, error bars represent the 95% confidence intervals (*p < 0.05, **p < 0.01, ***p < 0.001, no *p ≥ 0.05), and small points represent individual level data. Female measurements shown in panels A and C are redrawn in panel B and D, respectively.

When comparing the sexes at 25°C there is no effect of inversion genotype on proportion of time near food (*χ* ^2^ = 5.71, df = 2, *p* = 0.058; Table 1; Figure 3A), nor an effect of sex on proportion of time near food (*χ* ^2^ = 0.16, df = 1, *p* = 0.69), nor a sex‐genotype interaction (*χ* ^2^ = 0.24, df = 2, *p* = 0.89). The amount of time spent near the food was strongly impacted by presence of In(2L)t while comparing females across temperatures (*χ* ^2^ = 22.90, df = 2, *p* = 1.07e‐05; Table 2; Figure 3B). Female inverted flies spent less time near food than standard flies at 30°C (*t* = 3.09, df = 764.87, FDR corrected *p* = 0.013). Temperature had a significant impact on the proportion of time flies spent near food (*χ* ^2^ = 186.82, df = 1, *p* < 2e‐16), though we did not observe any gene‐by‐environment interaction (*χ* ^2^ = 0.42, df = 2, *p* = 0.81).

We also examined the proportion of time flies spent on the end of their enclosure away from food. When observing flies at 25°C, we observe that inverted homozygote flies are away from food significantly more than standard flies (*χ* ^2^ = 37.80, df = 2, *p* = 6.18e‐09; Table 1; Figure 3C). Female flies prefer the non‐food end of the enclosure more than males (*χ* ^2^ = 18.39, df = 1, *p* = 1.80e‐05), though there is no sex‐inversion interaction (*χ* ^2^ = 0.28, df = 2, *p* = 0.87). The preference for inverted homozygotic female flies for the far end of the enclosure is observed across temperatures (*χ* ^2^ = 78.01, df = 2, *p* < 2e‐16; Table 2; Figure 3D). Homozygous inverted flies spend more time away from food than standard at 20°C (*t* = −3.54, df = 641.73, FDR corrected *p* = 8.42e‐04; Figure 3D), 25°C (*t* = −6.09, df = 730.72, FDR corrected *p* = 1.10–08), and 30°C (*t* = −7.00, df = 646.21, FDR corrected *p* = 1.01e‐10). There is no observed effect of temperature on time spent away from food (*χ* ^2^ = 2.07, df = 1, *p* = 0.15), nor a temperature‐inversion interaction (*χ* ^2^ = 2.37, df = 2, *p* = 0.31).

Interestingly, Lee et al. 2017 also observed that In(2L)t is associated with variance in foraging within the DGRP (Lee et al. 2017). We reanalyzed their data and found that flies homozygous for In(2L)t were significantly worse at finding the food source, and subsequently starved sooner, than standard genotyped flies (*t* = 3.14, df = 19, FDR corrected *p* = 0.005; Table 3).

**TABLE 3:** Impact of In(2L)t genotype on foraging behavior, derived from a Student's T test on data from Lee et al. (2017).

Trait	*T* value	df	*p*
Survival dependent on foraging success	3.14	19	0.005

## 4 Discussion

In this study, we use automated behavioral observation to characterize how the inversion In(2L)t impacts multiple aspects of behavior in an environment and sex specific manner. By quantifying motion and activity of *D. melanogaster* from a set of genetically diverse crosses, we show that the presence of In(2L)t has an impact on time spent active (Figure 1A,B), the intensity of startle response (Figure 2), and the amount of time spent near and away from food (Figure 3). In(2L)t's impact on behavior is mostly consistent across temperatures, though we do note evidence of gene‐by‐environment interaction between temperatures for startle response intensity (Figure 2D). With these findings, we emphasize the importance of inversions as a model for investigating the genetic variation in complex traits such as behavior.

In(2L)t has a complex role in behavior with impacts on baseline and induced activity, and contains genes known to regulate aspects of behavior. In general, homozygous In(2L)t flies spend more time active than homozygous standard flies (Figure 1A), and more often venturing out from the food source (Figure 3D). Additionally, while In(2L)t flies may exhibit a reduced intensity of response to mechanical stimulus (Figure 2D), they seem to show little difference in time spent startled compared to the standard genotype (Figure 2C). One limitation of the study is that startle response behavior is observed regardless of whether the individuals were sleeping or awake, and therefore genetic differences in sleep behavior could influence startle response. There are several genes within In(2L)t that could explain the behavioral effects that we observe. The *foraging (for)* gene has a large impact on the range of travel while foraging (Nagle and Bell 1987; Pereira and Sokolowski 1993), and resides within In(2L)t. Indeed, previous work has identified that In(2L)t can explain part of the variance in foraging behavior (Lee et al. 2017), although that work failed to link variation at the *for* allele to foraging behavior. *Vglut* also resides within In(2L)t. Found near the 5′ In(2L)t breakpoint, this gene regulates transport of glutamate, and is associated with sleep and startle response (Birgner et al. 2010; Hamasaka et al. 2007). Alleles for these genes and others within the inversion may explain the impact of In(2L)t genotype on different aspects of behavior, and may themselves be linked due to suppressed recombination (Dobzhansky and Epling 1948; Kirkpatrick and Barton 2006).

The impact of In(2L)t and its environmental specificity is relevant due to the importance of understanding how changes in genetic architecture allow for seasonal adaptation. Season‐specific regulation of behavior is essential for successful adaptation to new environments (Chapman et al. 2015; Riters and Stevenson 2022). Strategies of inactivity versus activity are dependent upon the nutritional abundance (Wang et al. 2006), access to light (Welsh 1938), and temperature stressors (Buchholz et al. 2019) that come with changing seasons. For example, circadian control of activity, and conversely, the duration of sleep, is closely regulated by light and temperature, two elements of the environment that change from summer to winter (Boothroyd et al. 2007). There is strong evidence that natural variation in sleep exists across different environments, with night‐time duration of inactivity increasing at lower latitudes (Svetec et al. 2015). Given that the length of sleep can be selected for (Harbison et al. 2017), this indicates selection could be acting at different latitudinal environments on periods of activity, or induced activity, as in the case of stickleback fish altering their startle response intensity depending on the temperature of their local environment (Guderley et al. 2001). Selection acts on many aspects of behavior in different directions across different seasons, and this fluctuating directional selection may contribute to what allows genetic variation to persist.

The effect of In(2L)t on behavior is largely consistent across temperatures, helping to define In(2L)t's role in behaviors across seasons. Inverted genotype flies move more frequently than standard genotype flies across temperatures (Figure 2B), as well as being more likely to be away from food (Figure 3D). The consistent impact on behavior is relevant considering there is mounting evidence that In(2L)t is involved in the mediation of seasonal adaptation. The frequency of In(2L)t changes cyclically across the year, becoming highest in the winter months (Machado et al. 2021; Nunez et al. 2024). The seasonal fluctuations of In(2L)t frequency suggest that the phenotypic variance explained by In(2L)t could be under directional selection within a given season. Limited gene‐by‐environment effects can lead to a stable behavioral strategy and can potentially drive the yearly fluctuation in In(2L)t frequency if different behavioral strategies are beneficial at different times of the year. If higher activity is adaptive within colder months, then In(2L)t frequency would be higher, while high activity becoming maladaptive in warmer months could drive the In(2L)t frequency lower. The potential complex and shifting selection on the inversion can help explain both why In(2L)t frequency fluctuates seasonally (Machado et al. 2021) and how this inversion continues to be identified at intermediate frequency within populations (Nunez et al. 2024; Stalker 1980; van Delden and Kamping 1989). Future studies could build off the findings reported here by identifying the genetic regions within In(2L)t that are impacting different aspects of behavior and characterizing the fitness effects of high and low activity levels within seasonal environments.

## Author Contributions

**Benedict Adam Lenhart:** conceptualization (equal), data curation (equal), formal analysis (equal), funding acquisition (supporting), investigation (lead), methodology (equal), validation (lead), visualization (lead), writing – original draft (lead), writing – review and editing (equal). **Alan Olav Bergland:** conceptualization (equal), data curation (equal), formal analysis (equal), funding acquisition (lead), investigation (supporting), methodology (equal), project administration (lead), resources (lead), supervision (lead), visualization (supporting), writing – original draft (supporting), writing – review and editing (equal).

## Funding

We are supported by the NSF BIO‐DEB (EP) award # 2145688, NIH NIGMS award # R35GM119686 to AOB, start‐up funds provided by UVA to AOB, and by a fellowship from the Jefferson Foundation to BAL.

## Conflicts of Interest

The authors declare no conflicts of interest.

## Supporting information


**Table S1:** Lineage details for experimental crosses. This table includes the identity of the DGRP lineages used within this study, their In(2L)t genotype, as well as how the lineages were crossed to create the F1 offspring used in experiments.

## Data Availability

The DGRP lines used here are available from the Bloomington stock center in Indiana (https://bdsc.indiana.edu/). Inversion genotype tables for the lineages are all available from the DGRP website (https://quantgenet.msu.edu/dgrp/downloads.html). The reanalyzed phenotype data from previous studies can be found from their original publications. All other data is available for download at Dryad (https://doi.org/10.5061/dryad.8kprr4z42) and at Github (https://github.com/benedictlenhart/In_2L_t_behavior_Drosophila).
